# Evaluation of Recycled Spent Coffee Material Treated with Animal Glue, Starch, and Red Clay as Acid Materials

**DOI:** 10.3390/ma15196622

**Published:** 2022-09-23

**Authors:** Sung-Sik Park, Seung-Wook Woo, Jung-Shin Lee, Young-Mook Yun, Dong-Eun Lee

**Affiliations:** 1 Department of Civil Engineering, Kyungpook National University, 80 Daehakro, Bukgu, Daegu 41566, Korea; 2 Intelligent Construction Automation Center, Kyungpook National University, Global-plaza 905, 80 Daehakro, Bukgu, Daegu 41566, Korea; 3 School of Architecture, Civil, Environment and Energy Engineering, Kyungpook National University, 80 Daehakro, Bukgu, Daegu 41566, Korea

**Keywords:** spent coffee ground, red clay, waste recycling, construction material

## Abstract

Annual coffee consumption has increased to 10 million tons. Of the coffee consumed, 65% is discarded as spent coffee grounds (SCG). However, most SCG are buried in the ground as organic waste. The more coffee consumption increases, the more land is used for disposing of spent coffee. SCG recycling has gotten considerable attention as a solution involved in these issues. The construction community has studied means and methods to recycle SCG as construction materials, such as bricks, subgrade fillers, thermal insulators, etc. This paper presents a new method, which recycles SCG as a construction material, maximally using its acidity. The SCG were hardened with natural binders (i.e., animal glue (AG) and starch (S)) and red clay (RC). The SCG mixtures were pressed with 2 MPa in a cylindrical mold and cured for 7 days. Then, the strength, durability, and pH tests were measured. The AG- and RC-treated SCG sample, which outputs 1933 kPa of strength and a 4.9 pH value, is identified as the optimal sampling method among the acid materials produced in this study. The optimal sample decreases the pH to approximately 7 of water where 68% weight of Ordinary Portland cement was soaked in.

## 1. Introduction

Coffee is one of the most important foods in the world, and many kinds of coffee commodities are produced and distributed, including cookies, candy, bread, and beverages. The world’s annual coffee consumption has reached approximately 10 million tons, increasing by 1% of the compound annual growth rate between 2017 and 2021 [1]. By increasing coffee consumption, spent coffee grounds (SCGs) are projected to increase continuously. According to Murthy and Naidu, approximately 650 kg of spent coffee is obtained from 1 ton of green coffee beans [2], and approximately 6.5 million tons of SCG are expected annually. Generally, organic wastes, including SCG, are moved to landfills [3,4], but buried wastes can affect the ground environment and ecosystem of the land [5,6,7]. Therefore, researchers have been trying to find methods for waste recycling [8,9,10,11,12]. In particular, the growth in coffee consumption has attracted interest in SCG recycling.

SCG recycling has been studied in many areas. McNutt et al. investigated SCG utilization and discussed its potential as a bio-product [13]. SCG contains a variety of organic materials, and various methods are being developed for utilizing them. The recycled bio-products are used in many areas, such as biodiesel oil, combustible materials, food, and health. Additionally, SCG can be recycled and used as a material in the construction industry. Saberian et al. reviewed the recycling of SCG in the construction industry [14]. SCG was recycled as an additive to bricks, subgrade filler materials, thermal insulators, and sound-absorbing materials. Unlike previous studies, this study newly tried to apply SCG for pH reducing agent in construction site. Generally, the pH of the cement is not a major concern on structures, facilities, or buildings. However, in the case of underground structures or ground facilities, the high pH condition could affect to the ground system. A ground water which contacts with cement in underground caused an extremely alkaline leachate which has 13.5 of pH value [15], the value is similar with a bleach. In these cases of ground, some additive agents are treated to recover the alkaline ground condition. A traditional pH reduction agent of ground and soil is sulfur [16]. An elemental sulfur is oxidized in the ground by microbes to generate sulfate (SO_4_^2−^) and H^+^ ions [17]. However, sulfuric acid is one of the harmful materials and another, it could affect to the ground environmental system also. Thus, this study recycled the SCG.

A pH-reducing material was made with SCG to replace the sulfuric acid used in construction sites. To make an environment friendly cemented material, traditional adhesion binders were also used for making durable acid sample. In addition, a red clay of pH of 4–5 was also added for enhancing the neutralizing ability. The SCG and additive mixture was pressed under high pressure using animal glue and starch to prepare the sample, and the strength, durability index, and pH concentration were evaluated as basic neutralizing agents for cement leachate. To reduce the pH, red clay was also added to the sample. Recently, animal glue and starch adhesives have rarely been found in our daily lives because of the development of highly efficient adhesives. Many applications of both adhesives have been replaced by synthetic resin adhesives with good performance and low cost. However, in this study, organic additives (animal glue and starch) were used as binders to make the construction agent buried in the ground or concrete and create an eco-material made with natural components. In addition, animal glue has been applied to lime in previous studies. Ventola et al. found that animal glue increased the strength of lime mortar more than other organic additives [18], and Elert et al. studied the influence of animal glue on lime plasters. Starch was also applied as a clay binder [19]. Mansour et al. investigated starch-sandstone materials in the construction industry [20], and Kulshreshtha et al. used corn starch in their research [21].

## 2. Materials and Methods

### 2.1. Materials

#### 2.1.1. Spent Coffee Ground and Red Clay

**Figure 1:**
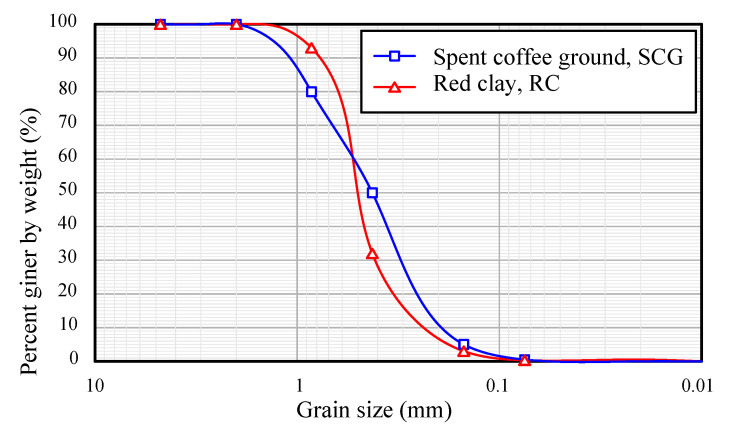
Particle size distribution of the sieved SCG and RC.

**Figure 2:**
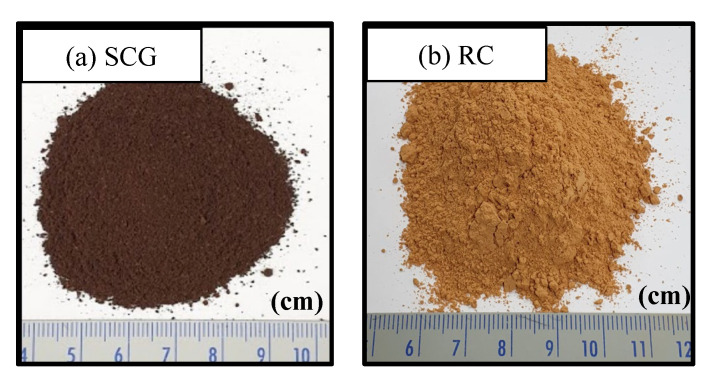
Optical photos of spent coffee grounds (**a**) and red clay (**b**).

SCG were obtained from the Starbucks in Daegu (Korea). Starbucks uses Arabica coffee beans and provides SCG to customers so that the extracted SCG can be recycled. The SCG collected underwent a dry chamber in a laboratory. The chamber maintained a low humidity of the interior and circulated the inner air. The dried SCG were sifted through a sieve with a mesh size of 2 mm and 0.15 mm. The geotechnical engineering properties of sieved SCG are listed in Table 1. The specific gravity (Gs) of SCG is known to be approximately 0.8–0.9, the pressure exerted on the coffee during brewing contributed to its density. In addition, SCGs are easily contracted when pressed. Thus, the density in Table 1 was calculated under the pressed condition with 2 MPa pressure. The coefficient of uniformity (Cu) and coefficient of curvature (Cc) were obtained from the sieve test. In this study, the Cu and Cc were 3.00 and 0.81, respectively. Figure 1 shows the grain-size distribution curve obtained from the sieve test, and Figure 2a shows the dried and sieved SCG.

Red clay (RC) was used in this study. RC has a higher iron ion content than other soils. The iron ion in RC makes the clay red. This study uses RC, which is obtained from Hwangtomyungga (Seoul, Korea). The RC is sieved so as to go through the mesh size of 2 mm and 0.15 mm as the SCG is. The chemical components of the RC and those of two other clays (i.e., bentonite and kaolinite) that are the most common clays in the world are presented in Table 2. The ferric oxide (Fe_2_O_3_) contents of the RC is 5 and 16 times higher than that of bentonite and of kaolinite, respectively. The chemical components of bentonite and those of kaolinite are accessible to other publications [22,23]. The particle size distribution of sieved red clay is shown in Figure 1. Figure 2b shows an optical photograph captured by a camera. The engineering properties are listed in Table 1. The specific gravity of red clay was 2.71, and its density was obtained under the compressed condition.

#### 2.1.2. Animal Glue and Starch

**Figure 3:**
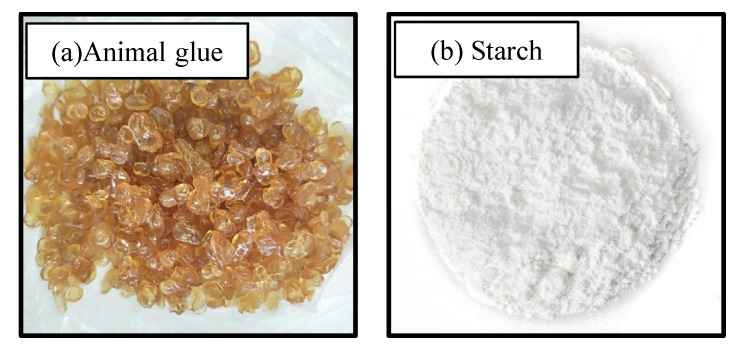
Optical photos of the animal glue (**a**) and starch (**b**).

Animal glue and starch were treated as aqueous solutions. Before mixing with SCG and red clay, water and binder were mixed in a 2:1 ratio, and the mixed solution was heated to a temperature between 60 and 70 °C to dissolve the glue. For the binder mixed with glue and starch, water, glue, and starch were prepared in a ratio of 2:0.5:0.5. Figure 3 shows the gelatin and starch used in this study.

### 2.2. Methods

#### 2.2.1. Sample Preparation

The SCG, red clay, and binder were mixed and placed in a cylindrical mold with a diameter of 5 cm. The mold was pressed at a constant pressure of 2 MPa until the height of the sample reached 5 cm. The ACONS Pro motorized automatic consolidation frame from VJ Tech (UK) was used as the pressing machine because the machine could press the sample at constant pressure and measure the height displacement while pressing the sample. The pressed sample was moved to a curing chamber, which maintained the temperature and humidity at 25°C and 70%, respectively. The compressed sample was transferred to a curing chamber where the temperature and humidity were maintained at 25 °C and 70%, respectively, and stored for 7 days. The samples were classified into AG (animal glue), S (starch), and AGS (animal glue and starch) based on the binder, and RC was attached to the sample containing red clay. The binder ratio was 15% of the SCG weight. Sample information is presented in Table 3.

#### 2.2.2. Test Methods

This study evaluated the strength, durability index, and pH. The strength was measured by performing an unconfined compression test according to ASTM D 2166 [24] and KS F 2314 [25]. The sample was pressed at a loading rate of 1 mm/min by moving the bottom plate upward while recording the compression distance and stress. The value of unconfined compressive strength (UCS) dictates the average of three specimens with an error of less than 5%. The durability index was measured using the slaking durability index according to Franklin and Chandara and ASTM D 4644 [26]. A drum containing ten samples was soaked in water and kept for one day. The soaked pieces were worn away while rotating the sieve drum at a speed of 20 rpm. The durability index was calculated by dividing the difference obtained by subtracting the dry weight of the rock pieces remaining after rotating the drum from the dry weight of the rock pieces before being soaked by the initial weight of the pieces. After conducting the test twice for each specimen, the durability index obtained in the second test was accepted as the representative value of the specimen according to ASTM D 4644 [26]. The pH was measured by placing 100 g of sample in 1 L of water. The pH of the sample placed in water was measured after 24 h. The pH meter used for the measurement was calibrated using a buffer solution of pH 7 before each test.

## 3. Results and Discussion

### 3.1. Test Result

**Figure 4:**
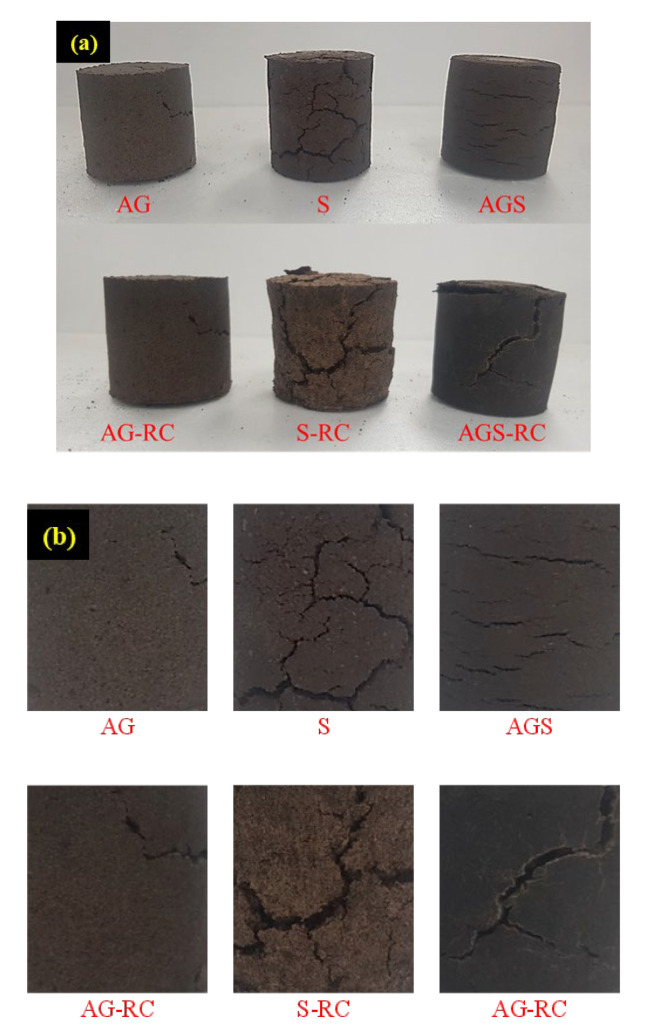
The photo of the cured samples (**a**) and surface of the each sample (**b**).

The final samples were cured in the chamber for 7 days and the surface of each sample is shown in Figure 4a,b, respectively. The outputs obtained by the tests are presented in Table 4. Some cracks occurred on the surfaces, and the samples that included starch and RC had more cracks than the others. The AG sample without starch and RC had the least fractures; in contrast, the AGS-RC sample had many fractures. The reason why fracture appears is that the moisture evaporates during the curing period, and the moisture in the binder solution led to the SCG contract as well. Being indicated by the height, diameter, and total unit weights of the cured samples shown in Table 4, the starch-included samples contract more rather than the animal glue samples do. In addition, the starch-included samples evaporate moisture more rather than the latter. The design unit weight and the binder weight were the same at sampling. However, the cured sample weights were different. The decrease in dimension and weight indicates evaporation of the moisture and SCG shrinkage.

**Figure 5:**
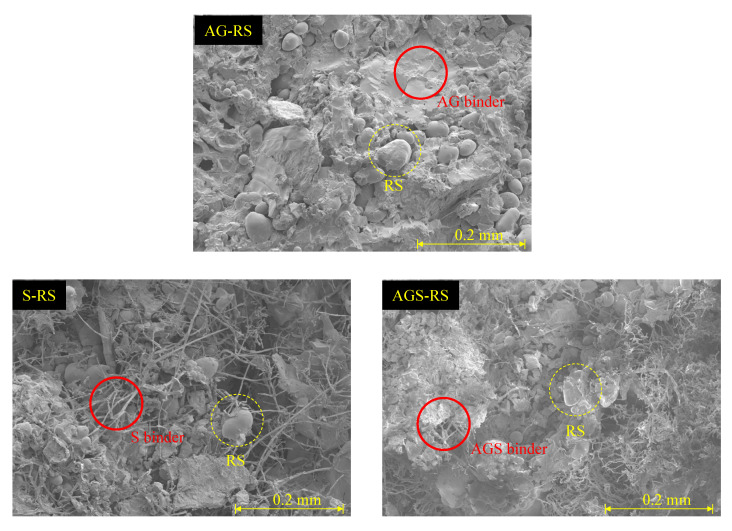
Scanning electrochemical microscopy photo of the AG-RC, S-RC, and AGS-RC samples.

To analyze the binding matrix, scanning electrochemical microscopy (SEM) was performed and SEM analysis was performed on the AG-RC, S-RC, and AGS-RC samples to observe all the additives used in this study; Figure 5 shows the SEM photo. The AG binder covered all areas of the voids between the SCG and RC. However, there were many voids in the S-RC photo. The S binder formed a needle-shaped thin skeleton. In addition, RC and SCG were not covered by the binder. The cracks on the S sample were due to the different skeletons of the AG and S binders. The AG binder absorbed water well, whereas the moisture of the binder evaporated. The water content was measured by heating the cured sample to over 100 °C. All samples had a water content of less than 1%; thus, it was considered that the curing time was sufficient for hardening and drying.

### 3.2. Unconfined Compressive Strength (UCS)

**Figure 6:**
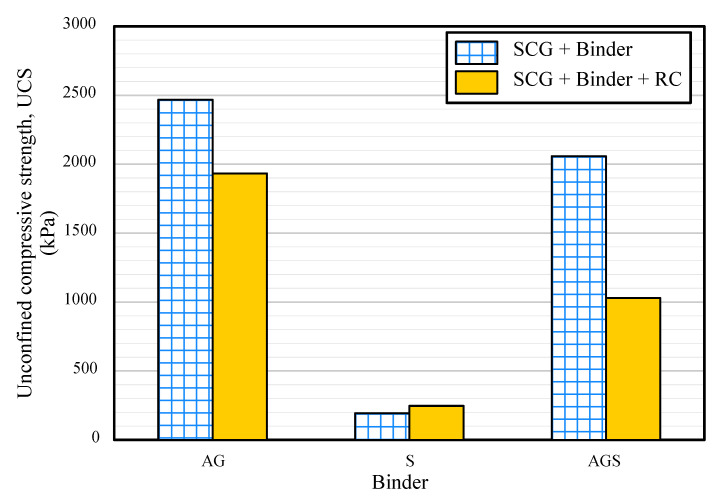
Unconfined strength of the samples.

Figure 6 shows the UCS of each sample. The UCS exhibited different values depending on the binder. The animal glue sample showed a strength of up to 2468 kPa, whereas the starch sample showed a poor strength of 193 kPa. In addition, the strengths decreased when RC was added to the sample regardless of the binder; the UCS of AGS decreased by half by adding RC. At construction sites, cement is the most common binder, and the strength of cement-treated clay has been reported to be approximately 1500–3000 kPa in many studies. It is expected that AG, AGS, and AG-RC samples can support the bearing capacity of the ground when they are buried in the cement-treated ground as an aggregate because their UCS is approximately 2000 kPa or more. However, the S, S-RC, and AGS-RC samples are difficult to apply to the ground because of their poor strength. It is considered that this structure of the starch binder decreases the strength, and that of the AG binder increases the strength. Thin skeletons of the starch binder shown in Figure 5 are not strong enough to withstand high pressures and many void-derived stress concentrations in the binder structure. In contrast, the AG binder has a few voids and the binder skeleton is widely wrapped around the SCG and RC so that stress is transmitted throughout the entire sample.

### 3.3. Slake Durability Index

**Figure 7:**
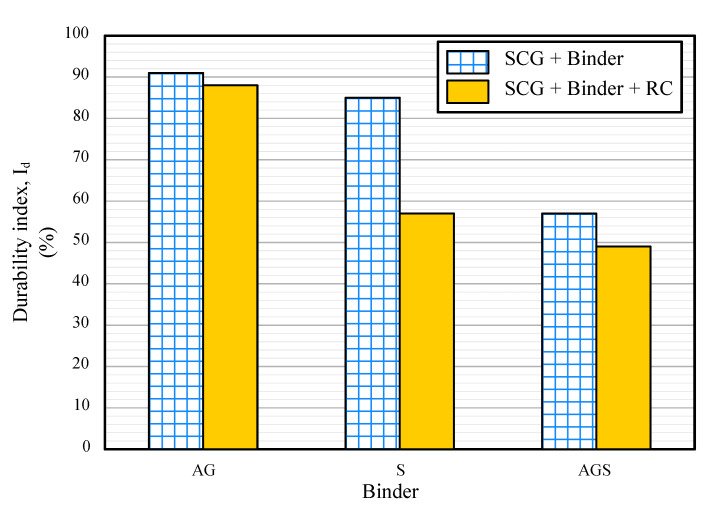
Slake durability index of the samples.

Figure 7 shows the slake-durability index (Id) of each sample. The Id values showed a similar pattern to that of the UCS. The durability of the AG sample was high, and its value decreased with the inclusion of S and RC. This is because, in general, the lower the strength, the lower the durability. Franklin and Chandara categorized the samples based on the Id; values above 75% were classified as very high and above 90% as extremely high. The AG and AG-RC samples were classified as extremely high and very high, respectively. S, AGS, and AGS-RC were medium, and S-RC was very low. Based on the strength and durability test results, only the AG and AG-RC samples could be used as construction materials. The other samples did not have sufficient strength characteristics.

### 3.4. pH Value

**Figure 8:**
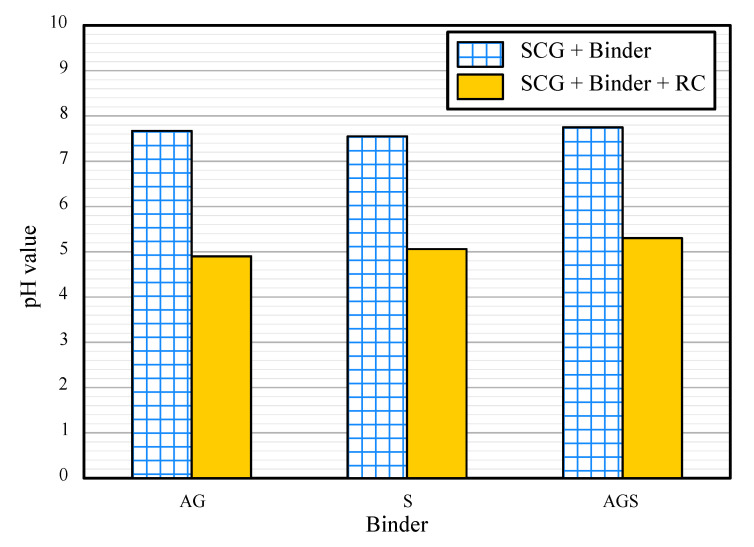
pH values of the samples.

**Figure 9:**
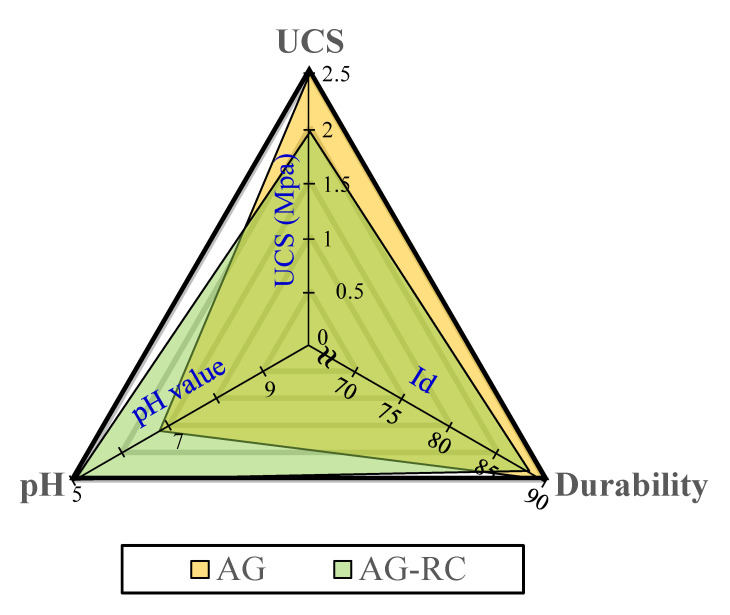
Comparison of the test result of AG and AG-RC sample.

Figure 8 shows the pH values of the AG, S, and AGS samples which are approximately 7. In general, the pH of SCG is between 5 and 6 [27]. However, binder-treated SCG showed a higher value than normal. It is considered that the pH result increases from the pH of binders because animal glue and starch binder showed alkali characteristics according to other studies [28]. However, acidic behavior was observed for samples containing red clay. All the RC samples showed low pH values (<6). In particular, the highest acidity was obtained for the AG-RC sample with a pH of 4.9. Overall, the UCS, durability, and pH test results of the AG binder-treated SCG were better than those of the S and AGS samples. Figure 9 shows a comparison of the performance of the AG and AG-RC samples. Although the AG sample showed good strength and durability, the pH results were not acidic. The UCS and durability of the AG-RC sample were slightly lower than those of the AG sample, but it was an acid neutralizer at the construction site, indicating that the AG-RC sample is the best SCG recycling method compared to the other samples in this study.

### 3.5. pH Reduction of the Cement Water

**Figure 10:**
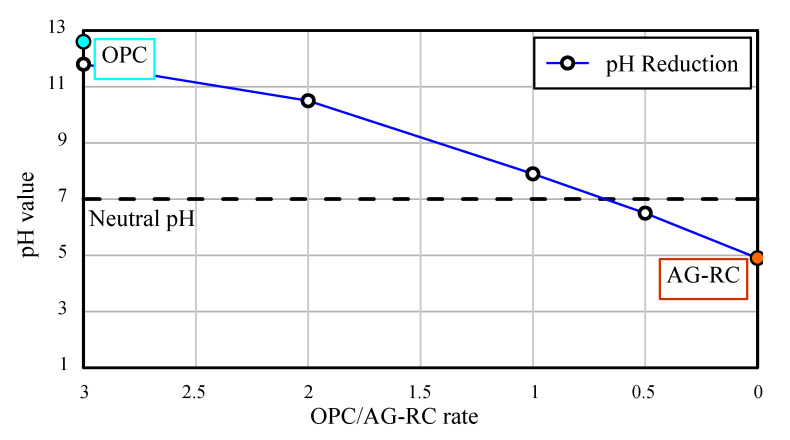
pH reduction effects of AG-RC sample.

The pH reduction effect of the AG-RC sample was evaluated by soaking it in water containing cement powder. The 100 g of AG-RC samples were soaked in four beakers containing 1 L of water. Ordinary Portland cement (OPC) was added to the AG-RC-soaked waters. OPC weighing 50, 100, 200, and 300 g were added to the beaker, and the pH was measured after 24 h. In addition, the pH of the OPC was measured using the same method as the other SCG samples. The measurement results are presented in Table 5. The OPC/AG-RC rate is defined by the weight ratios of the OPC to the AG-RC. The relationship between the pH values and the OPC/AG-RC rate is presented in Figure 10. It is confirmed that the more the pH of the OPC decreases, the more the OPC/AG-RC rate decreases. The OPC is neutralized at pH 7, at which the OPC/AG-RC rate is between 1 and 0.5. The pH reduction line crosses the neutral pH line at the OPC/AG-RC rate of 0.68. It means that the OPC would have a neutral pH at 68% of the weight of the AG-RC sample, given the SCG recycled agent used in this study is applied to a construction site. Especially, if the AG-RC sample is used as an aggregate buried around an underground structure or facility, it may work as a hardened aggregate that supports the ground bearing capacity and neutralizes the pH of the groundwater around the structure as well.

## 4. Conclusions

This study attempted to evaluate the SCG recycling material, which included a natural binder and red clay. The SCGs were mixed with the binder and RC, and then strength, durability, and pH tests were conducted. The following results were obtained from the tests:

✔ The animal glue-treated SCG sample showed 2468 kPa of UCS, and red clay addition did not enhance the UCS. The starch binder and red clay formed numerous cracks on the surface of the sample, and the starch and red clay samples showed a poor UCS of approximately 193 kPa. It was considered that the difference in the binding structure of binders leads to the strength result.
✔ The best slake durability test index was 91% for the animal-binder-treated sample. This value can be classified as an extremely high grade. The durability index showed a trend similar to that of the UCS results. The starch binder and the red soil reduced this value. Both the treated samples had the poorest index.
✔ AG, S, and AGS samples showed a pH value of approximately 7. The value decreased further with the addition of RC. The highest acid value was observed for the AG-RC samples (4.91). With a UCS of 1933 kPa, slake durability index of 89%, and acidic pH, AG-RC was the most suitable for the goal of this study.
✔ The pH value of Ordinary Portland cement was 12.6, and the AG-RS sample could neutralize the cement at a 68% weight rate. In particular, if AG-RS samples are produced as aggregates and buried around underground structures or facilities, it may reduce the generation of high alkaline groundwater which occurs when groundwater contact with underground cement-based structures.

## Figures and Tables

**Table 1:** Geotechnical properties of the sieved SCG and RC.

Materials	Pressed Density (t/m^3^)	Coefficient of Uniformity, Cu	Coefficient of Curvature, Cc
Spent coffee ground, SCG	118	3.00	0.81
Red clay, RC	235	2.82	1.29

**Table 2:** Chemical components of the red clay.

Clay	Color	Component (%)							
		SiO_2_	Al_2_O_3_	K_2_O	Fe_2_O_3_	TiO_2_	MgO	CaO	L.O.I
Red clay	Red	48.63	25.74	4.93	16.91	0.74	0.62	2.13	0.30
Bentonite	Gray	61.28	17.79	1.24	3.01	-	2.10	4.54	10.04
Kaolinite	White	46.70	36.70	1.25	1.09	0.05	0.05	0.01	14.30

**Table 3:** Sample making table.

ID	Clay Material	Binder	Binder Ratio	Red Clay Ratio	Curing Time
AG	SCG	Animal glue	15 %	-	7 days (148 h)
S		Starch			
AGS		Animal glue and Starch			
AG-RC	SCG and Red clay	Animal glue		20%	
S-RC		Starch			
AGS-RC		Animal glue and Starch			

**Table 4:** Test results of all samples.

ID	Height (mm)	Diameter (mm)	Total Unit Weight (kN/m^3^)	Water Content (%)	UCS (kPa)	Slake Durability Index, Id	pH Value
AG	4.95	4.97	11.81	0.84	2468	91	7.67
S	4.51	4.62	9.21	0.54	247	85	7.55
AGS	4.83	4.79	10.72	0.61	2058	57	7.75
AG-RC	4.97	4.99	14.36	0.94	1933	88	4.9
S-RC	4.73	4.71	13.10	0.64	193	57	5.06
AGS-RC	4.95	4.96	12.31	0.51	1029	49	5.3

**Table 5:** pH reduction test results.

AG-RC (g)	OPC (g)	Water (L)	OPC/AG-RC Rate *	pH Value
0	100	1	-	12.6
100	0		0	4.9
	50		0.5	6.5
	100		1.0	7.9
	200		2.0	10.5
	300		3.0	11.8

* OPC/AG-RC rate: weight rate of AG-RC and OPC.

## Data Availability

Data sharing is not applicable to this article, as no datasets were generated or analyzed during the current study.
